# Eukaryotic translation initiation factor 6 overexpression plays a major role in the translational control of gallbladder cancer

**DOI:** 10.1007/s00432-019-03030-x

**Published:** 2019-10-04

**Authors:** Nicole Golob-Schwarzl, Christina Wodlej, Florian Kleinegger, Margit Gogg-Kamerer, Anna Maria Birkl-Toeglhofer, Johannes Petzold, Ariane Aigelsreiter, Michael Thalhammer, Young Nyun Park, Johannes Haybaeck

**Affiliations:** 1 Diagnostic and Research Institute of Pathology, Medical University of Graz, Graz, Austria; 2 Institute of Dermatology and Venerology, Medical University of Graz, Graz, Austria; 3 Center for Biomarker Research in Medicine, Graz, Austria; 4 Department for Biomedical Research, Core Facility Alternative Biomodels and Preclinical Imaging, Medical University of Graz, Graz, Austria; 5 Department of General Surgery, Medical University of Graz, Graz, Austria; 6 Department of Pathology, Yonsei University, College of Medicine Soul, Seoul, South Korea; 7 Department of Pathology, Medical Faculty, Otto-von-Guericke-University, Leipziger Straße 44, 39210 Magdeburg, Germany; 8 Department of Neuropathology and Molecular Pathology, Medical University of Innsbruck, Innsbruck, Austria

**Keywords:** Gallbladder cancer, Eukaryotic translation initiation factor 6, Biomarker

## Abstract

**Background:**

Gallbladder cancer (GBC) is a rare neoplasia of the biliary tract with high mortality rates and poor prognosis. Signs and symptoms of GBC are not specific and often arise at late stage of disease. For this reason, diagnosis is typically made when the cancer is already in advanced stages, and prognosis for survival is less than 5 years in 90% of cases. Biomarkers to monitor disease progression and novel therapeutic alternative targets for these tumors are strongly required. Commonly, dysregulated protein synthesis contributes to carcinogenesis and cancer progression. In this case, protein synthesis directs translation of specific mRNAs, and, in turn, promotes cell survival, invasion, angiogenesis, and metastasis of tumors. In eukaryotes, protein synthesis is regulated at its initiation, which is a rate-limiting step involving eukaryotic translation initiation factors (eIFs). We hypothesize that eIFs represent crossroads in the development of GBC, and might serve as potential biomarkers. The study focus was the role of eIF6 (an anti-association factor for the ribosomal subunits) in GBC.

**Methods:**

In human GBC samples, the expression of eIF6 was analyzed biochemically at the protein (immunohistochemistry, immunoblot analyses) and mRNA levels (qRT-PCR).

**Results:**

High levels of eIF6 correlated with shorter overall survival in biliary tract cancer (BTC) patients (*n* = 28). Immunohistochemical data from tissue microarrays (*n* = 114) demonstrated significantly higher expression levels of eIF6 in GBC compared to non-neoplastic tissue. Higher eIF6 expression on protein (immunoblot) and mRNA (qRT-PCR) level was confirmed by analyzing fresh frozen GBC patient samples (*n* = 14). Depletion of eIF6 (using specific siRNA-mediated knockdown) in Mz-ChA-2 and TFK-1 cell lines inhibited cell proliferation and induced apoptosis.

**Conclusion:**

Our data indicates that eIF6 overexpression plays a major role in the translational control of GBC, and indicates its potential as a new biomarker and therapeutic target in GBC.

**Electronic supplementary material:**

The online version of this article (10.1007/s00432-019-03030-x) contains supplementary material, which is available to authorized users.

## Background

Gallbladder cancer (GBC) is the most common neoplasia of the biliary tract and is characterized by high mortality rates and poor prognosis. Early symptoms are vague and anatomically the gallbladder lacks a serosa to limit the spreading of cancer, so the diagnosis of GBC frequently occurs at an advanced stage. The 5-year survival rate is less than 5% for more advanced stages. Over 80% of GBC cases are adenocarcinomas and originate from the fundus (Shaffer 2008). GBC is the second leading cause of cancer death among women, with a mortality rate only slightly lower than that of breast cancer (Leal et al. 2013).

GBC shows distinct geographic variations. High incidence and mortality rates are seen in selected such as Central European countries, American-Indian and Chilean-Mapuche. The tumor spreads to the liver and adjacent organs, and is distributed by blood, lymphatics, and the peritoneum (Sharma et al. 2017; Zhang et al. 2017; Shaffer 2008). More effective strategies for the treatment of GBC are essential to improve the prognosis for GBC patients (Wu et al. 2007; Leal et al. 2013). Therefore, an understanding of the molecular mechanisms of GBC is necessary to develop novel therapeutic alternatives.

Protein translation can be divided into four steps: initiation, elongation, termination and ribosome recycling. Translation is mainly regulated at the initiation step, and dysregulation leads to abnormal gene expression, possibly resulting in uncontrolled cell growth (Sonenberg and Hinnebusch 2009). The translation initiation process is monitored by eukaryotic initiation factors (eIFs). They might serve as tumor suppressors or promote carcinogenesis and tumor progression in different types of cancer (Spilka et al. 2013; Silvera et al. 2010). The eIF signaling cascade is mainly regulated via the PI3 K/AKT/mTOR pathway due to its pivotal role in regulating cell growth and proliferation. This pathway operates as sensor, for instance, for nutrition/energy availability and adapts gene expression, ribosome biogenesis, and protein translation to surrounding conditions of the cells. Uncontrolled/hyperactivated PI3K/AKT/mTOR signaling leads to dysregulated protein synthesis, which contributes to carcinogenesis and cancer progression (Golob-Schwarzl et al. 2017; Jackson et al. 2010; Spilka et al. 2013; Engelman 2009; Liu et al. 2009). Protein translation regulation mainly takes place at the initiation phase which is also the rate-limiting step of protein synthesis in eukaryotes. The initiation starts with the formation of the 43S ribosome pre-initiation complex, consisting of the 40S small ribosomal subunit, methionine tRNAi and various eIFs, followed by the recruitment of the 43S ribosome complex to the 5´UTR region of the mRNA via the cap-binding complex eIF4F (Golob-Schwarzl et al. 2017; Spilka et al. 2013; Silvera et al. 2010). The eIF4F complex comprises the scaffold protein eIF4G, the cap-binding protein eIF4E, and the ATP-dependent helicase eIF4A.

The last step of translation initiation is the formation of the mature 80S ribosome by joining the 60S subunit. To prevent untimely binding of the 60S ribosomal subunit to the 40S ribosomal subunit, the 25 kDa protein eIF6 plays an important role as the anti-association factor. Since no pseudogenes nor gene duplications of eIF6 can be found, it can be proposed that there is a strong evolutionary pressure for tight control of the protein expression and concentration, underlying the importance of proper functioning of eIF6 (Parsyan et al. 2011; Parsyan 2014). eIF6 binds to 60S ribosomal subunits already in the nucleus and releases them into the cytoplasm after phosphorylation by mitogens or growth factors (Golob-Schwarzl et al. 2017; Spilka et al. 2013). Therefore, it can be found in the nucleus and in the cytoplasm. Approximately 30% of eIF6 are localized in the nucleus, and 70% of eIF6 are located in the cytoplasm (Zhu et al. 2017). As interaction of the 60S with the 40S subunit is impaired by eIF6, translation initiation is blocked (Spilka et al. 2013; Zhu et al. 2017). Many studies state that eIF6 is rate limiting in the regulation of translation (Golob-Schwarzl et al. 2017; Finch et al. 2011; Gartmann et al. 2010; Miluzio et al. 2011; García-Márquez et al. 2015). Furthermore, eIF6 also plays an important role in the biogenesis of the 60S ribosome (Golob-Schwarzl et al. 2017; Spilka et al. 2013).

Dysfunction of translation control is probably the endpoint of oncogenic pathways that support cellular transformation and tumor development (Silvera et al. 2010). However, most of these pathways are hyperactivated and pro-oncogenic in tumors (Parsyan et al. 2011; Loreni et al. 2013). eIF6 was found to be overexpressed in various cancer types, like colorectal cancer, ovarian serous carcinoma, acute promyelocytic leukemia, non-small cell lung cancer and in head and neck cancer (Golob-Schwarzl et al. 2017; Spilka et al. 2013; Parsyan et al. 2011; Gantenbein et al. 2018).

In this study, we investigated the involvement of various eIF subunits, focusing on eIF6 in GBC. For this purpose, we used analyses like immunohistochemistry and immunoblotting to characterize the eIF6 protein expression levels. qRT-PCR was used for gene expression studies, as well as The Cancer Genome Atlas dataset to correlate gene expression with overall survival. Finally, we assessed the therapeutic potential of targeting eIF6 by performing siRNA-mediated knockdown experiments in two biliary tract cancer (BTC) cell lines (TFK-1, Mz-ChA-2).

## Methods

### Ethics statement

The collection and use of human-derived gallbladder specimens were approved by the Ethics Committee of the Medical University of Graz, Austria, according to the ethical guidelines of the 1975 Declaration of Helsinki (28-294 ex 15/16) and the Institutional Review Board of the Severance Hospital (no. 4-2014-0421, Seoul, South Korea). All samples and medical data used in this study were irreversibly anonymized and their clinical and pathological data are listed in Tables 1 and 2.

**Table 1:** Clinical and pathological characteristics of patients assessed as TMA tissue specimens

GBC	Number (*n* = 114)	%
Gender		
Female	70	61.4
Male	44	38.6
Median age	65	
Stage		
I	13	11.4
II	40	35.1
II IA	9	7.9
III B	16	14
IV A	6	5.3
IV B	23	20.2
Unknown	7	6.1
Grading		
1	41	36
2	53	46.5
3	20	17.5
Intensity (cytoplasmic)		
0	50	43.9
1	50	43.9
2	5	4.4
3	9	7.8
Intensity (nuclear)		
0	93	81.6
1	18	15.8
2	3	2.6
3	0	0
Density (cytoplasmic)		
0%	50	43.9
0–10%	0	0
11–49%	0	0
50–79%	0	0
80–100%	64	56.1
Density (nuclear)		
0%	93	81.6
0–10%	0	0
11–49%	0	0
50–79%	0	0
80–100%	21	18.4

**Table 2:** Clinical and pathological characteristics of 26 patient cryo samples

Adenocarcinoma of the gallbladder	Number *(n* = 14)	%
Gender		
Female	9	64.3
Male	5	35.7
Age (± SD)	70.4 (8.5)	
Subtype adenocarcinoma		
Adenosquamous	4	28.6
Tubulary	4	28.6
Mucinous	1	7.1
Tubulo-papillary	4	28.6
Mixed	1	7.1
Grading		
1	7	50
2	6	42.9
3	1	7.1

NNT	*n* = 12	%
Gender		
Female	2	16.7
Male	10	83.3
Age (± SD)	57.8 (8.1)	

### Tissue microarrays (TMAs)

114 formalin-fixed, paraffin-embedded patient samples were retrospectively collected from the University Hospital of Seoul. Hematoxylin–eosin-stained (H/E) slides were reviewed by an experienced, board-certified pathologist (J.H.), who confirmed the diagnoses and identified the areas of tumor and non-neoplastic tissue for each tissue microarray core. The usage of patient samples for the generation of TMAs was approved by the local ethics committees (No. 28-294 ex 15/16 and 4-2014-0421). Tissue cores of 1.2 mm in diameter were punched out from the chosen tumor areas and embedded as TMA in a fresh paraffin block according to a specific pattern. Tissue sections were cut (4 µm) and placed on adhesive-coated glass slides followed by immunohistochemical analysis. Clinical and pathological data are listed in Table 1.

### Immunohistochemistry (IHC)

The protein expression of eIF6 was analyzed by immunohistochemical staining. IHC was performed on a Ventana Immunostainer XT (Ventana Medical Systems, Tucson, USA) by heat-induced epitope retrieval (HIER) in cell conditioning solution for 30 min. For detection, the ultra-VIEW universal DAB Detection Kit (Ventana Medical Systems, Tucson, USA) was used. Each core was semi-quantitatively scored, and the intensity score was assessed as follows: 0 = no staining, 1 = weak staining, 2 = moderate staining and 3 = strong staining. Additionally, stained tumor cells were recorded in a percentage ranging from 0 to 100% (TIS).

### Human gallbladder cancer patient samples

Human GBC samples and non-neoplastic tissue (NNT) were obtained at the Diagnostic and Research Institute of Pathology, Medical University of Graz, Austria or the BioBank Graz, Austria. Informed consent was obtained from all patients. All tissue samples were acquired during surgery, immediately snap frozen in liquid nitrogen, and stored at − 80 °C until protein or RNA extraction. The cohort description is listed in Table 2.

### Protein extraction and immunoblot

For generation of protein lysates, frozen tissue samples were homogenized with a MagNA Lyser homogenizer (Roche Diagnostics, Risch-Rotkreuz, Switzerland) and lysed in Nonidet-P40-based lysis buffer (0.05 M Tris–HCl, 0.15 M NaCl, 0.5% NP-40, 0.1 mM Pefabloc, 1 mM DTT, cOmplete Mini EDTA-free, PhosSTOP). Protein concentrations were determined using Bradford protein assay (BioRad Protein Assay Dye Reagent; BioRad Laboratories GmbH, Munich, Germany). 30 µg of total protein lysate were loaded onto sodium dodecyl sulfate (SDS)-polyacrylamide gels (30% Acrylamide/Bisacrylamid solution; ROTH, Karlsruhe, Germany), subjected to electrophoresis in mini-vertical electrophoresis units (Hoefer Inc, Richmond, USA), and blotted onto PVDF membranes (Immobilin-P Transfer Membrane; Millipore, Massachusetts, USA) using a Semi-Dry Blotting Unit (SCIE-PLAS; Cambridge, England). The membranes were blocked with 5% non-fat dried milk (AppliChem; Darmstadt, Germany) in TBS supplemented with 0.1% Tween (TBST) for 1 h at room temperature. The primary antibody eIF6 (1:1000, #PA5-31066, Thermo Fischer Scientific Inc., Massachusetts, USA) was diluted in TBST containing 5% BSA and incubated at 4 °C overnight. The membranes were washed with TBST, followed by incubation with a horseradish peroxidase-conjugated secondary antibody (anti-rabbit 1:5000; GE Healthcare Life Science, Buckinghamshire, UK). Proteins were visualized using the chemiluminescence Amersham ECL Western Blotting Detection Reagent (GE Healthcare Life Science, Buckinghamshire, UK) by the Image Quant LAS 500 (GE Healthcare Life Science, Buckinghamshire, UK) detection system. Signals were normalized to the glyceraldehyde-3-phosphate dehydrogenase (GAPDH) loading control (mAb dilution 1:1000, #2118, Cell Signaling, Frankfurt, Germany). Densitometrical analysis was performed using ImageJ software.

### Quantitative real-time PCR (qRT-PCR)

Total RNA was isolated from snap-frozen human GBC tissue and NNT using TRIzol Reagent (Life Technologies; Woolston, UK) followed by phenol–chloroform extraction. siRNA-transfected cells were washed three times with PBS, scraped off with PBS and lysed with TRIzol Reagent. Total RNA (1 µg) was reversely transcribed using the High-Capacity cDNA Reverse Transcription Kit (Applied Biosystems, Foster City, USA) according to the manufacturer´s instructions (GeneAmp 9700 Thermocycler, Applied Biosystems; Foster City, USA). For gene expression analyses, the Power SYBR Green PCR Master Mix Kit (Applied Biosystems, Foster City, USA) was used in a QuantStudio™ 7 Flex Real-Time PCR System (Applied Biosystems, Foster City, USA). *GAPDH* was found to be the most stable endogenous control using NormFinder, and relative gene expression levels were calculated using the 2^∆∆CT^ method.

### Cell culture

The cell line TFK-1 was cultured in RPMI-1640 supplemented with 10% FBS and antibiotics (penicillin 100 U/ml; streptomycin 100 µg/ml). Mz-ChA-2 cells were cultured in RPMI-1640 supplemented with 10% FBS, 2 mM l-glutamine, 1 × MEM non-essential amino acids (Gibco, Life Technologies, Darmstadt, Germany) and antibiotics (penicillin 100 U/ml; streptomycin 100 µg/ml).

Both cell lines were cultured at 37 °C in a humid atmosphere with 5% CO_2_ and passaged when 90% confluency was reached (use of Trypsin–EDTA 0.05%, Life Technologies, California, USA). To exclude mycoplasma contamination, both cell lines were routinely checked using PromoKine PCR Mycoplasma Test Kit (Biomedica Medizinprodukte GmbH & Co KG, Vienna, Austria). STR profiling (PowerPlex 16HS System, Promega, Madison USA) was done to verify cell lines. Both cell lines (TFK-1 and Mz-ChA-2 cells) were obtained from the American Type Culture Collection (ATCC).

### siRNA transfection

For *eIF6* knockdown, two target sequences were used: *eIF6*-*1* (20 nM, Hs_ITGB4BP_5, 5′-CTGCTTTGCCAAGCTCACCAA-3′, #SI0309633, QIAGEN, Hilden, Germany) and *eIF6*-*2* (20 nM, HS_ITGB4BP_6, 5′-CTGGTGCATCCCAAGACTTCA-3′, #SI03099768 QIAGEN, Hilden, Germany). A scrambled siRNA (SC) construct (20 nM, Allstars negative control siRNA #1027280, QIAGEN, Hilden, Germany) was used as negative control. Transfection experiments were performed using Metafectene^®^SI^+^ transfection reagent (Biontex, Munich, Germany) according to the manufacturer´s instructions. For the transfection 1x SI buffer, Metafectene^®^ SI + and siRNA were mixed in 6-well plates. After an incubation of 15 min at room temperature, 1x10^5^cells were added to each well. Cells with transfection mix were cultured at 37 °C in a humidified atmosphere of 5% CO_2_. Cells were harvested after incubation for 48 h and 72 h. Three independent experiments were performed.

### MTT assay

Transfected cells and controls were seeded in 96-well plates (1 × 10^4^ cells/well) and cultivated without antibiotics for 48 h and 72 h. Metabolic activity of cells was determined on the basis of mitochondrial conversion of 3-(4,5-dimethylthiazol-2-yl)-2,5-diphenyltetrazolium bromide (MTT, Sigma-Aldrich, Missouri, USA) to insoluble formazan. Therefore, cells were incubated with 5.5 mg/ml MTT for 2 h at 37 °C. The supernatant was discarded, and cells were lysed with 3% SDS. Formazan crystals were dissolved in 0.05 M isopropanol/HCl for 15 min at room temperature under vigorous shaking. Absorption was measured at 570 nm (Synergy™4, BioTek, Winooski, USA). Each sample was carried out in sixfold determination, and three independent experiments were performed.

### Apoptosis

Apoptotic cells were detected using YO-PRO™-1 (Thermo Fisher Scientific, Massachusetts, USA) reagent. siRNA-transfected and control cells were seeded in 96-well plates (1x10^4^ cells/well). After 48 h and 72 h cells were incubated with YO-PRO™-1 for 15 min at 37 °C, the supernatant was removed, and cells were washed with PBS. After excitation (485 nm), emission was measured at 535 nm. Each assay was performed in sixfold determination, and three independent experiments were carried out.

### Colony formation assay

Transfected cells and controls were seeded into six-well plates (500 cells/well) and cultivated over 2 weeks. The medium was changed every 3 days. After cultivation, cells were washed three times with PBS followed by fixation in 4% paraformaldehyde (Sigma-Aldrich, Missouri, USA). Fixed cells were stained with freshly prepared Giemsa solution (1:10 with ddH_2_O) (Sigma-Aldrich, Missouri, USA) for 20 min. Afterwards, cells were rinsed with distilled water; colonies were analyzed using an inverse microscope (Nikon TMS—Inverted Microscope, Tokyo, Japan). Three independent experiments were carried out.

### Statistical analysis

The Cancer Genome Atlas (TCGA) public dataset including 28 CCC subjects was analyzed to identify the association between gene expression stratified by the median and survival. Kaplan–Meier curves were generated using the survival R package. The log-rank test was applied to test for association of survival and gene expression. All results were expressed as mean ± standard deviation (SD). Differences between groups were assessed using Student’s *t* test or Mann–Whitney *U* test based on data distribution. Results of the cell culture experiments were statistically tested using one- or two-way ANOVA with Bonferroni post-test. A *p* value < 0.05 was considered as statistically significant. Statistical analysis and graph generation were performed using GraphPad PRISM version 5.0 (GraphPad software Inc., La Jolla, CA, USA).

## Results

### eIF6 is a marker of gallbladder cancer (GBC) with bad prognosis

**Fig. 1:**
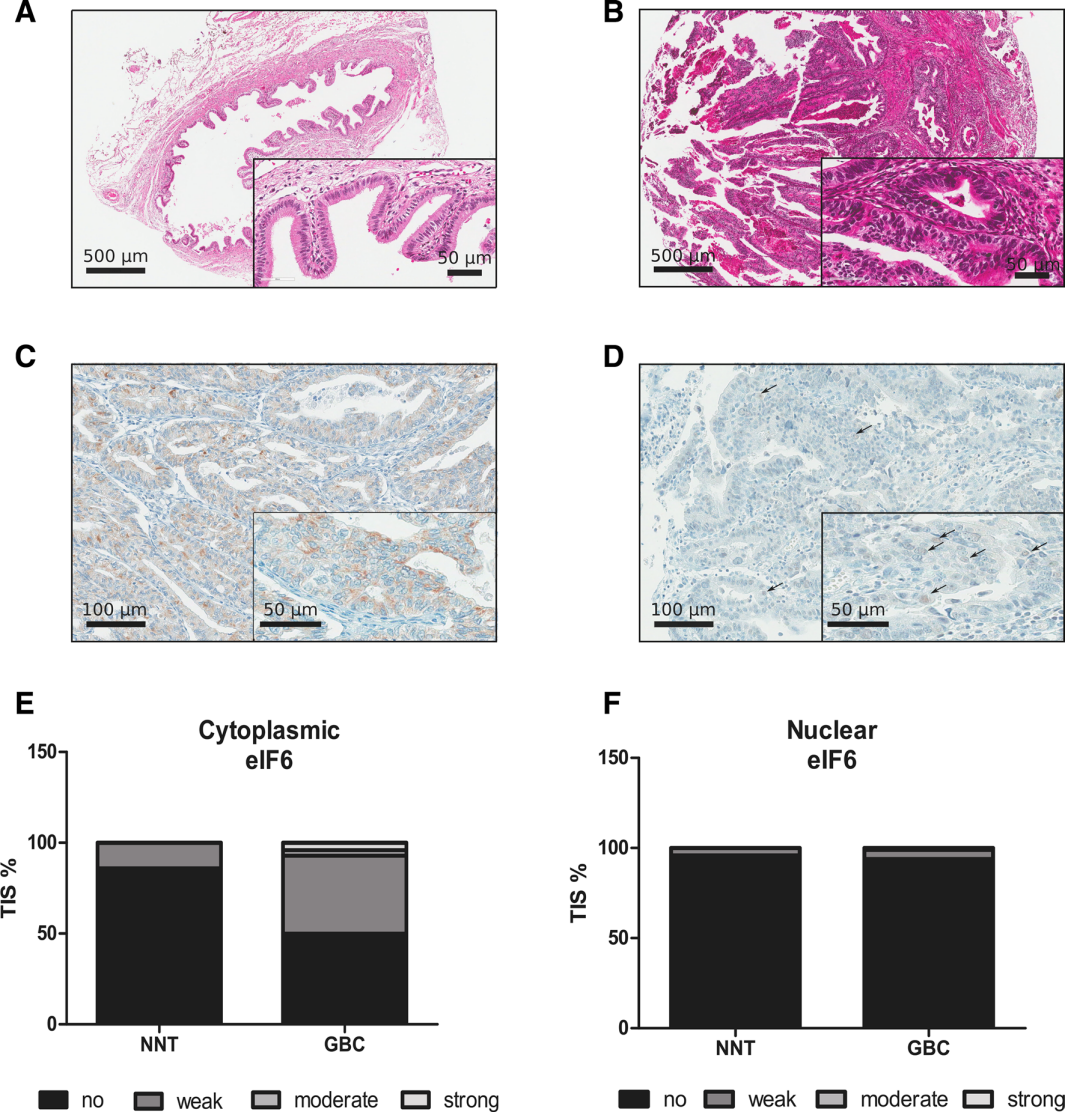
eIF6 is overexpressed in GBC compared to NNT. **a** Representative hematoxylin–eosin (H/E) staining was reviewed to confirm the diagnoses and to identify the areas of formalin-fixed, paraffin-embedded non-neoplastic tissue (NNT) for each tissue microarray core. Scale bars: 500 µm and 50 µm. **b** Representative hematoxylin–eosin (H/E) staining was reviewed to confirm the diagnoses and to identify the areas of formalin-fixed, paraffin-embedded tumors for each tissue microarray core. Scale bars: 500 µm and 50 µm. **c** Representative IHC pictures of eIF6-stained non-neoplastic tissue (NNT). eIF6 is mainly located in the cytoplasm of NNT GBC tissue. eIF6 is evident in both the nucleolus and cytoplasm of tumor cells. Scale bars: 500 µm and 50 µm. **d** Representative IHC picture of nuclear eIF6-stained GBC tissue. eIF6 is evident in both the nucleolus and cytoplasm of tumor cells. Scale bars: 500 µm and 50 µm. **e** Tissue intensity scores (TIS) revealed stronger staining intensity of eIF6 in the cytoplasm in GBC patients compared to NNT. **f** Tissue intensity score (TIS) revealed no changes in the staining intensity of eIF6 in the nucleolus in GBC patients compared to NNT

We determined eIF6 expression levels in patient-derived GBC tissue and NNT by immunohistochemical staining (IHC) of tissue microarray (TMA) sections to address the prognostic potential of eIF6 (Fig. 1a–d). Clinical data of patients analyzed by IHC are listed in Table 1. 114 GBC patient samples were analyzed, and respective adjacent NNT served as controls. eIF6 staining was mainly observed in the cytoplasm but also in the nucleus (Fig. 1c, d). The tissue intensity score (TIS) of cytoplasmic eIF6 was higher in GBC tissue in the cytosol compared to NNT (Fig. 1e, f). However, there were no changes regarding eIF6 immunoreactivity manifested in the nucleus compared to NNT (Fig. 1e, f).

**Fig. 2:**
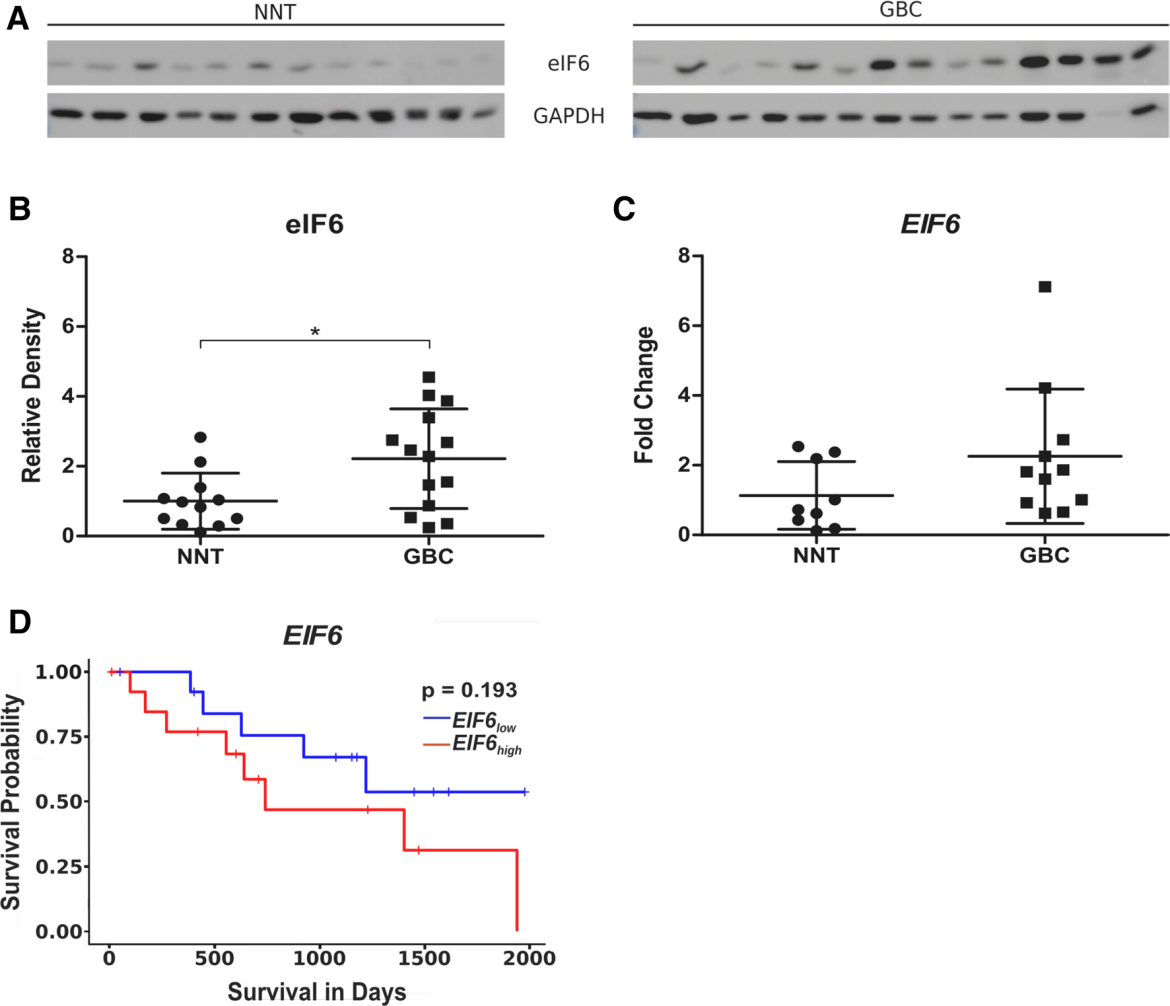
eIF6 expression is increased in GBC compared to NNT. **a** Representative immunoblots of eIF6 protein expression in fresh frozen GBC samples compared to NNT. **b** Densitometrical analysis of fresh frozen GBCs (*n* = 14) proved the significantly increased expression of eIF6 in tumor tissue compared to NNT (*p < 0.05). The intensity of the bands was normalized to GAPDH, which served as loading control. Due to Gaussian distribution of data, Student’s *t* test was performed for statistical analysis. **c** qRT-PCR of EIF6 mRNA was performed in fresh frozen 11 GBC and fresh frozen 9 NNT samples. Fold change values of EIF6 normalized to GAPDH as housekeeping gene are depicted. Bars represent mean ± SD. *p < 0.05, **p < 0.01, ***p < 0.001. Statistical analysis: Mann–Whitney *U* test. **d** High expression is highlighted in red and low expression in blue. Kaplan–Meier curves represent the correlation between EIF6 gene expression and survival of BTC patients based on TCGA database an in silico analysis stratified by the median. Statistical analysis: log-rank test (*p* = 0.193)

To examine whether eIF6 protein levels, compared to NNT, are also upregulated in cryo material of gallbladder tumor tissue, total protein lysates of 14 GBC tissue specimens and 12 non-neoplastic gallbladder tissue specimens were prepared. The patients’ characteristics for the cryo material are listed in Table 2. These lysates were analyzed by immunoblotting and revealed overexpression of eIF6 in GBC samples (Fig. 2a) when compared to NNT. Densitometric evaluation (eIF6/GAPDH) of all samples analyzed by ImageJ is outlined in Fig. 2b and revealed significantly higher expression of eIF6 compared to NNT (*p* < 0.05). This patient cohort was also analyzed for *EIF6* mRNA expression levels using qRT-PCR which revealed differences (*p* = 0.1106) (Fig. 2c). These data suggest that eIF6 may play an important role during tumorgenesis of GBC.

### High expression of eIF6 correlates with shorter survival and poor prognosis of BTC patients

Based on the results shown above, we investigated the correlation between the expression of eIF6 and patient overall survival. Therefore, an in silico analysis including 28 patients of The Cancer Genome Atlas (TCGA) database was performed. Tests for statistical significance were carried out with the log-rank test. Kaplan–Meier curves were generated to assess a potential association of *EIF6* expression with overall survival in biliary tract cancer (BTC) patients. Higher expression of *EIF6* in BTC was associated with a poorer overall survival compared to low expression of *EIF6* (*p* = 0193) (Fig. 2d).

Additionally, other eIF subunits (e.g., *1, 1AX, 1B, 2α, 3A, 3C, 3H, 5, 4E, 4G1, 4G2, and 4G3*) were investigated for their influence on BTC patients’ overall survival (Supplementary Figure S1 and S2). Higher expression of eIF4E in BTC showed a significant poorer overall survival compared to low expression of eIF4E (*p* = 0.040) (Supplementary Figure S2A). There was no significant difference for the *eIF* subunits *1, 1AX, 1B, 2α, 3A, 3C, 3H, 5, 4G1, 4G2 and 4G3*. These findings suggest that eIF6 might have a different functional role and might serve as a novel prognostic marker for the overall survival of BTC patients.

### In vitro knockdown of eIF6 reduces cell growth and proliferation, and increase apoptosis

Based on the results of the basic characterization of GBC patient-derived tissue (Figs. 1, 2), eIF6 was identified as a novel factor significantly overexpressed in GBC, which is why this protein might represent a potential target for future therapeutic interventions.

**Fig. 3:**
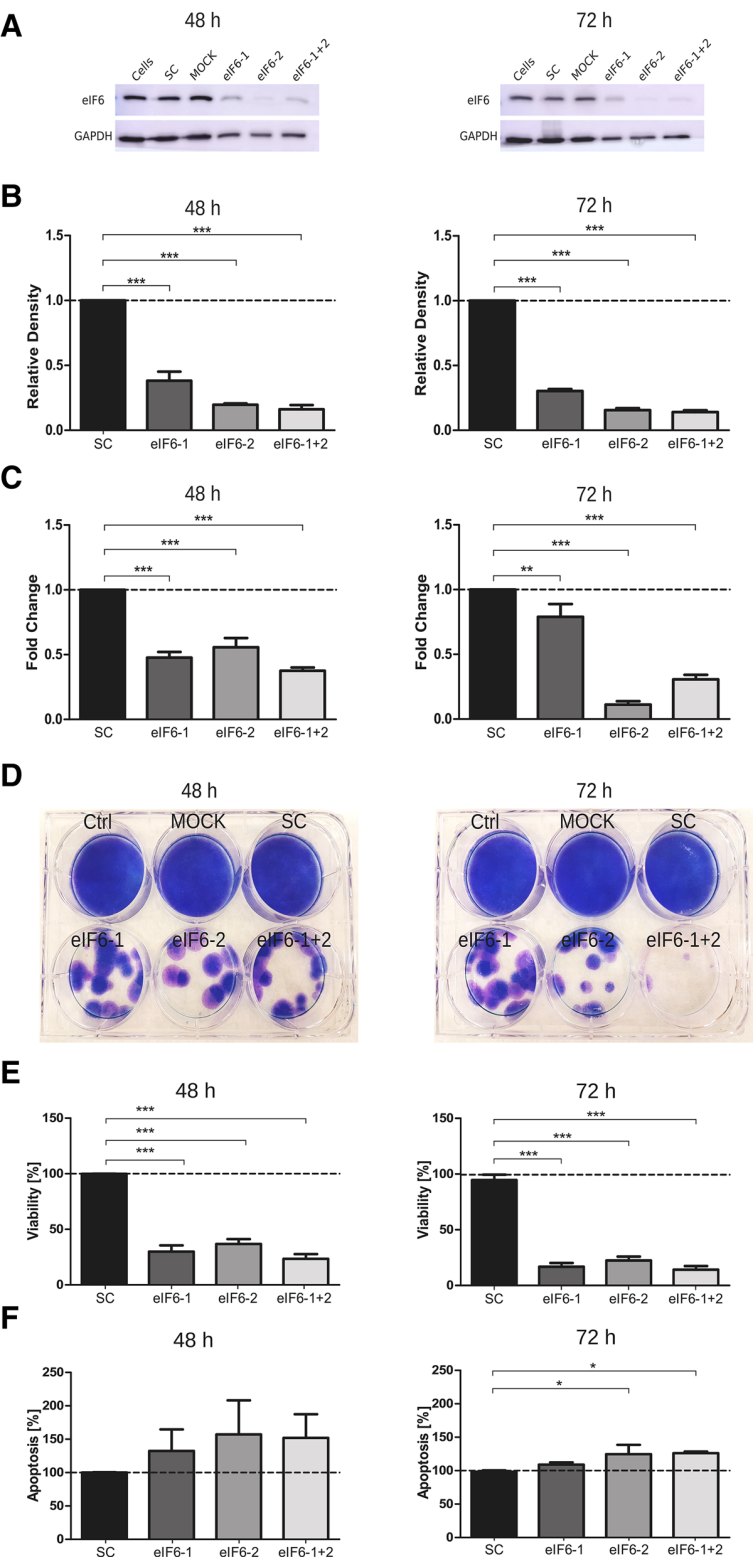
Knockdown of eIF6 in Mz-ChA-2 cell line. **a** Representative immunoblots of successful knockdown of eIF6 with siRNA after 48 and 72 h in Mz-ChA-2 cells. **b** Densitometrical analysis of eIF6 signals normalized to GAPDH, which served as loading control. In Mz-ChA-2 cells, eIF6 protein levels are significantly (***p < 0.001) decreased after 48 and 72 h, compared to scrambled siRNA-transfected condition. **c** mRNA levels of *EIF6* in transfected Mz-ChA-2 cells analyzed by qRT-PCR and normalized to *GAPDH* mRNA levels. Three independent experiments were carried out. Bars represent mean ± SD. *p < 0.05, **p < 0.01, ***p < 0.001. Statistical analysis: one-way ANOVA with Bonferroni post-test. **d** Representative picture of colony formation assay of induced eIF6 knockdown after 48 h and 72 h post-transfection in Mz-ChA-2 cell line. **e** Cell viability of Mz-ChA-2 cells transfected with eIF6 siRNA after 48 h and 72 h (***p < 0.001). **f** Graphs show apoptosis rates after eIF6 knockdown compared to SC after 48 h and 72 h (**p < 0.001). Three independent experiments were carried out. Bars represent mean ± SD. *p < 0.05, **p < 0.01, ***p < 0.001. Statistical analysis: one-way ANOVA with Bonferroni post-test

**Fig. 4:**
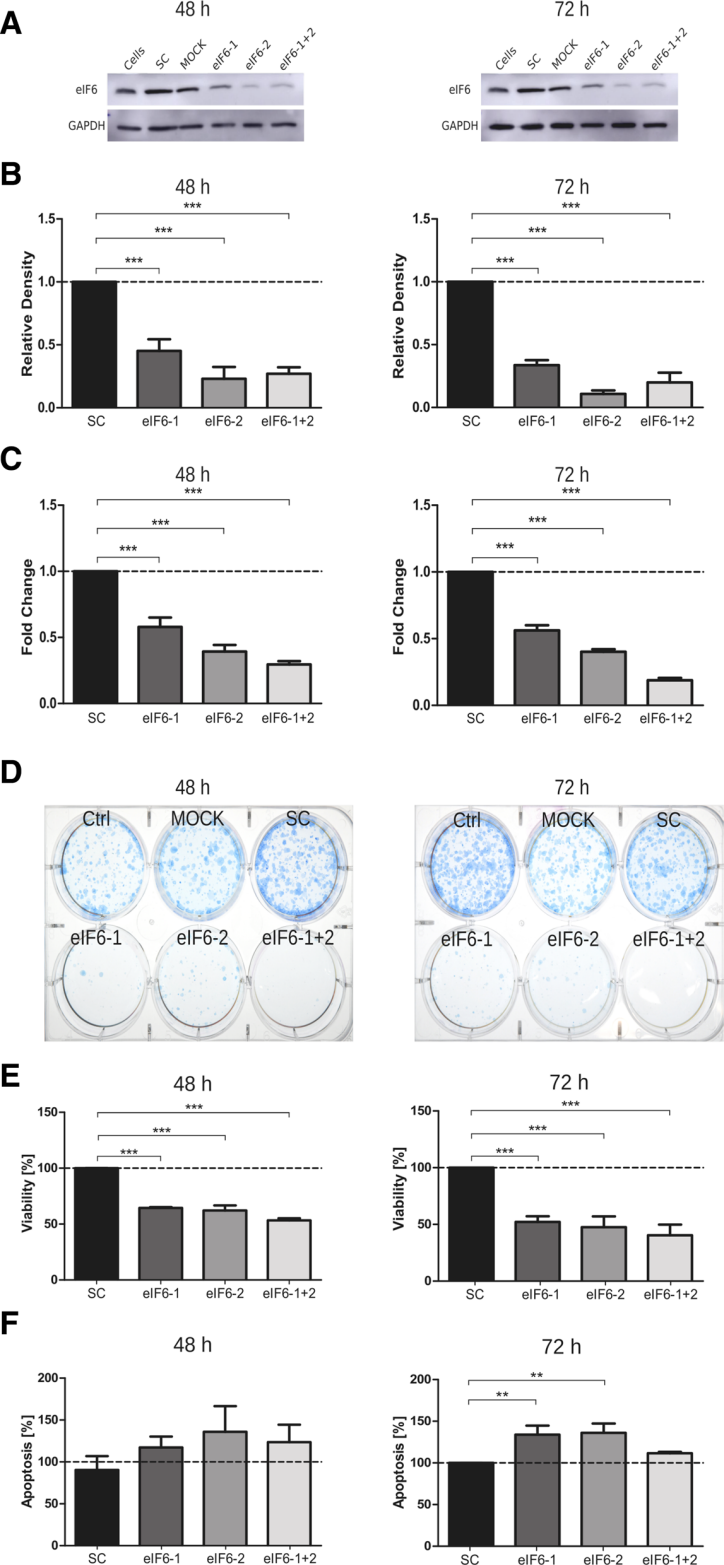
Knockdown of eIF6 in TFK-1 cell line. **a** Representative immunoblots of successful knockdown of eIF6 with siRNA after 48 and 72 h in TFK-1 cell line. **b** Densitometrical analysis of eIF6 signals normalized to GAPDH, which served as loading control. In TFK-1 cells, eIF6 protein levels are decreased after 48 and 72 h post-transfection, compared to scrambled siRNA-transfected condition. **c** mRNA levels of *EIF6* in transfected TFK-1 cells analyzed by qRT-PCR and normalized to *GAPDH* mRNA levels. Three independent experiments were carried out. Bars represent mean ± SD. *p < 0.05, **p < 0.01, ***p < 0.001. Statistical analysis: one-way ANOVA with Bonferroni post-test. **d** Representative colony formation assay of eIF6 knockdown induced 48 h and 72 h post-transfection in TFK-1 cell line. **e** Cell viability of TFK-1 cells transfected with eIF6 siRNA after 48 h and 72 h (***p < 0.001). **f** Graphs show apoptosis rates after eIF6 knockdown compared to SC after 48 h and 72 h (**p < 0.001). Three independent experiments were carried out. Bars represent mean ± SD. *p < 0.05, **p < 0.01, ***p < 0.001. Statistical analysis: two-way ANOVA with Bonferroni post-test

To evaluate the consequences of reduced eIF6 levels, Mz-ChA-2 and TFK-1 cell lines were transfected with eIF6 targeting siRNA. Knockdown efficiency was evaluated by immunoblot 48 and 72 h post-transfection. eIF6 knockdown was highly efficient (between 60 and 80% at both time points analyzed) in Mz-ChA-2 cells independent of whether the siRNA constructs were used individually (eIF6-1 and eIF6-2) or in combination (eIF6-1 + 2) (Fig. 3a). In TFK-1 cells, single siRNA constructs and the siRNA pool reduced eIF6 levels to 60–80% compared to non-targeting scrambled siRNA (Fig. 4a). Densitometrical evaluations of eIF6 knockdown in both cell lines are shown in Figs. 3b and 4b.

Additionally, knockdown efficiency was evaluated by qRT-PCR 48 and 72 h post-transfection (Figs. 3c, 4c). Analysis of *eIF6* mRNA levels in Mz-ChA-2 and TFK-1 cells after silencing revealed a reduction of about 70–80% compared to non-targeting scrambled siRNA (SC). The mRNA levels of *eIF6* in Mz-ChA-2 and TFK-1 cells were reduced by 50–70% compared to SC after 72 h after transfection (Figs. 3c, 4c).

Colony formation assay after 48 and 72 h eIF6 knockdown was investigated with Mz-ChA-2 and TFK-1 cells (Figs. 3d, 4d). Compared to control cells (cells, MOCK and SC), both transfected cell lines evidently had fewer colonies after 3 weeks of cultivation.

In Mz-ChA-2 and TFK-1 cells, cell viability was significantly (*p* < 0.005) reduced in comparison to transfection with SC (Figs. 3e, 4e). After 72 h of transfection in Mz-ChA-2 and TFK-1, cell viability was significantly reduced by 70% by all eIF6 targeting constructs compared to SC control (Figs. 3e, 4e).

Conversely, the apoptosis rates of Mz-ChA-2 and TFK-1 cells were increased by targeting eIF6 compared to transfection with SC after 48 and 72 h (Figs. 3f, 4f*)*. After 72 h of transfection in Mz-ChA-2 and TFK-1, apoptosis was significantly increased by 20–40% in all siRNA constructs compared to SC (Figs. 3f, 4f).

## Discussion

Gallbladder cancer is a rare neoplasia of the biliary tract that differs from other cancers of the digestive tract. Signs and symptoms of GBC are not specific and often arise late in the clinical course of disease. For this reason, diagnosis is typically made when the cancer is already in advanced stages, and prognosis for survival is less than 5 years in 90% of cases. Furthermore, GCB is characterized by its resistance to radio and chemotherapy (Randi et al. 2006). Biomarkers to monitor disease progression and novel therapeutic alternative targets for these tumors are strongly required.

eIFs are involved in the translation of growth factors, proteins influencing cell cycle, and growth, as well as apoptosis and malignant transformation. mTOR pathway members and eIFs are overexpressed in malignancies, such as squamous cell carcinoma of the head and neck, cancer of the lung, thyroid, breast and other cancer types (Wang et al. 2012; Gantenbein et al. 2018). However, data on eIFs in GBC are still limited.

The in silico analysis of The Cancer Genome Atlas (TCGA) database revealed a significant influence of eIF4E on overall survival of BTC patients. eIF4E is part of the eIF4F complex, interacts with the mRNA and facilitates the recruitment of the 40S ribosomal subunit. mTOR directly phosphorylates the 4E-binding protein (4E-BP), which are inhibitors of eIF4E, to relieve translational suppression. Hyperactivation of the mTOR pathway occurs in majority of the cancers, which results in increased eIF4E activity. Thus, translational control via eIF4E acts as a convergence point for hyperactivation and promotes tumorigenesis (Siddiqui and Sonenberg 2015). Targeting eIF4E is currently under investigation in many cancer types and might represent a target in GBC therapy (Siddiqui and Sonenberg 2015). eIF4E seems to be an important part of the tumorigenesis GBC patients.

eIF6 affects the maturation of 60S ribosomal subunits (Sanvito et al. 2000) as well as formation of the 80S ribosome as anti-association factor (Miluzio et al. 2009), and can be found in the nucleolus as well as in the cytoplasm. eIF6 was found to be overexpressed in various cancer types, like metastatic colorectal cancer (CRC) (Golob-Schwarzl et al. 2017) or non-small cell lung cancer (Gantenbein et al. 2018). eIF6 expression was reported to limit cell growth and transformation (Basu et al. 2001). Regulation of eIF6 is neither controlled transcriptionally via c-Myc nor post-transcriptionally via the PI3 K/AKT/mTOR signaling (Chendrimada et al. 2007). The specific molecular mechanisms underlying the role of eIF6 in these processes remain unclear (Parsyan et al. 2011; Siddiqui and Sonenberg 2015; Gantenbein et al. 2018; Miluzio et al. 2011; García-Márquez et al. 2015; Sanvito et al. 2000; Biffo et al. 1997; Sanvito et al. 1999).

Our study is the first to give evidence that eIF6 is overexpressed in GBC compared to non-neoplastic gallbladder tissue, and that it is a predictor for overall survival in GBC. Genetic interference with eIF6 by RNAi technique reduced cell proliferation and induced apoptosis in vitro. Our results highly suggest that eIF6 might be an important biomarker in GBC. Therefore, in pathologic examinations of the gallbladder, RNA or protein quantification of eIF6 could possibly predict the survival of GBC patients (Golob-Schwarzl et al. 2017; Sanvito et al. 2000; Miluzio et al. 2015; Flavin et al. 2008; Rosso et al. 2004). Thus, directly targeting eIF6 by reducing its expression or inhibiting its activity might improve therapeutic efficacy.

eIF6 overexpression was found in human CRC but not in non-neoplastic tissue, indicating a potential new therapeutic target (Golob-Schwarzl et al. 2017; Sanvito et al. 2000). Patients with ovarian serous adenocarcinomas showed eIF6 overexpression and a correlation with patients’ overall survival (Golob-Schwarzl et al. 2017; Flavin et al. 2008). TCGA data set displayed an influence of eIF6 on patients’ overall survival in GBC. Moreover, the IHC staining of GBC for eIF6 revealed its overexpression in the cytoplasm of gallbladder tumor cells, whereas staining intensity and density were less in non-neoplastic gallbladder tissue. This increase in cytoplasmic eIF6 levels in human FFPE tissue specimens was already reported in CRC, ovarian serous adenocarcinoma, and pleural mesothelioma (Golob-Schwarzl et al. 2017; Miluzio et al. 2015; Flavin et al. 2008). However, there was no change in the eIF6 expression in the nucleus when comparing GBC tissue and the non-neoplastic cells.

Mortality of a total knockout of eIF6 was distinguished in a mouse model for MYC-stimulated lymphomagenesis where littermates of eIF6^−/−^ mice were not viable. In eIF6^+/−^ heterozygous knockout mice, tumor-free survival was observed, suggesting that eIF6 might limit tumor progression (Gartmann et al. 2010). In *Saccharomyces cerevisiae,* depletion of *TIF6*, the yeast homologue for eIF6, led to reduced cell proliferation and viability (Basu et al. 2001).

Since eIF6 turned out to be a novel promising target on protein and mRNA level for GBC, we performed knockdown experiments to investigate this factor in more detail. After successful knockdown of eIF6, the proliferation rate and the colonogenicity of TFK-1 and Mz-ChA-2 cells were significantly reduced. This confirms previously published data, where after *eIF6* knockdown eIF6 was significantly reduced in HCT116 cells and led to reduced proliferation and colonogenicity (Golob-Schwarzl et al. 2017). Many cellular phenomena were related to alteration in activities of cap-dependent and internal ribosomal entry site (IRES)-driven translation during programmed cell death. Even though activated, eIF4F cooperates with c-MYC to promote malignant transformation. This effect is mediated by the power of eIF4F to block MYC-induced apoptosis through translational activation of negative regulators of the apoptotic machinery (Lin et al. 2009; Ruggero 2009; Polunovsky et al. 1996; Wendel et al. 2004). In our present study, we also analyzed apoptosis levels after knockdown of eIF6, and revealed a significant increase after 48 h and 72 h in transfected TFK-1 and Mz-ChA-2 cells. However, the nucleolar cytoplasmic relevance and function of eIF6 in, as well as its contribution to, tumorigenesis raise questions that are difficult to answer. Hence, the relevance of eIF6 in tumorigenesis seems to be context dependent and remains to be fully elucidated.

## Conclusion

In conclusion, we suggest that eIF6 might serve as a prognostic biomarker for overall survival in GBC patients, and that its regulation could serve as a potential new therapy approach in GBC. eIF6 can might be used in the future as a marker in immunohistochemistry. Still, there is a need for future investigations of eIF6 expression in GBC to determine whether eIF6 drives gallbladder carcinogenesis.

Nevertheless, still much effort for further investigation on eIF6 in GBC needs to be taken. Consequently, the aim of our study was to define novel therapeutic targets or potential new biomarkers to facilitate diagnosis and to improve the dismal prognosis associated with this highly malignant disease.

## Electronic supplementary material

Below is the link to the electronic supplementary material.

Fig. S1: Clinical relevance of *EIF1, EIF1AX, EIF1B, EIF2A, EIF3A, EIF3C, EIF3Hf* and E*IF5* gene expression on BTC patients` overall survival using TCGA data set. (A-H) Kaplan–Meier curves comparing the median *EIF1* (*p* = 0.204)*, EIF1AX* (*p* = 0.388)*, EIF1B* (*p* = 0.903)*, EIF2A* (*p* = 0.543)*, EIF3A* (*p* = 0.906)*, EIF3C* (*p* = 0.887)*, EIF3H* (*p* = 0.593), and E*IF5* (*p* = 0.821) gene expression and BTC patients´ overall survival (*n* = 28) of TCGA database an in silico analysis. High expression is highlighted in red and low expression in blue. (PDF 489 kb)

Fig. S2: Clinical relevance of *EIF4E, EIF4G1, EIF4G2,* and *EIF4G3* patients’ overall survival using TCGA data set. (A–D) Kaplan–Meier curves comparing the median *EIF4E* (*p *= 0.040)*, EIF4G1* (*p* = 0.288)*, EIF4G2* (*p* = 0.820), and *EIF4G3* (*p* = 0.936) gene expression and overall survival of BTC patients´ of TCGA dataset an in silico analysis for BTC (*n* = 28). High expression is highlighted in red and low expression in blue. (PDF 248 kb)

## Abbreviations

AKT: Protein kinase B
BSA: Bovine serum albumin
BTC: Biliary tract cancer
eIF: Eukaryotic translation initiation factors
FFPE: Formalin-fixed paraffin-embedded tissue
GAPDH: Glyceraldehyde 3-phosphate dehydrogenase
GBC: Gallbladder cancer
IHC: Immunohistochemistry
IRES: Internal ribosomal entry site
MTT: 3-(4,5-Dimethylthiazol-2-yl)-2,5-diphenyltetrazolium bromide
mRNA: Messenger RNA
mTOR: Mechanistic target of rapamycin
NNT: Non-neoplastic tissue
PBS: Phosphate-buffered saline
PI3 K: Phosphatidylinositol-4,5-bisphosphate 3-kinase
qRT-PCR: Quantitative real-time PCR
SDS: Sodium dodecyl sulfate
siRNA: Small interfering RNA
TBST: Tris-buffered saline–Tween
TCGA: The Cancer Genome Atlas
TIS: Tissue intensity score
TMA: Tissue microarrays
