# Supplementary figures and images for: Eukaryotic translation initiation factor 6 overexpression plays a major role in the translational control of gallbladder cancer
Source: J Cancer Res Clin Oncol. 2019 Oct 4;145(11):2699–711. doi: 10.1007/s00432-019-03030-x (PMC6800842; doi:10.1007/s00432-019-03030-x)

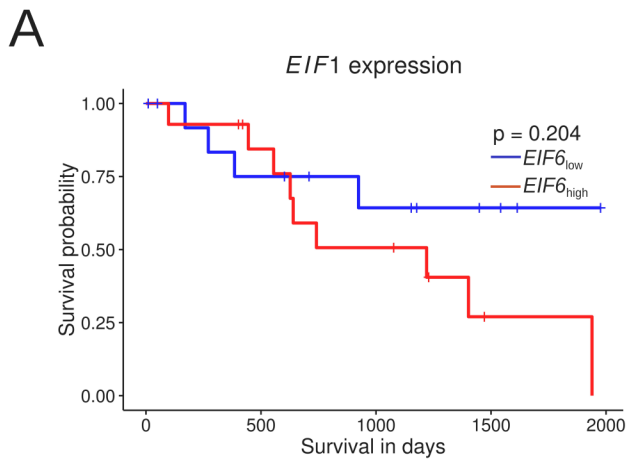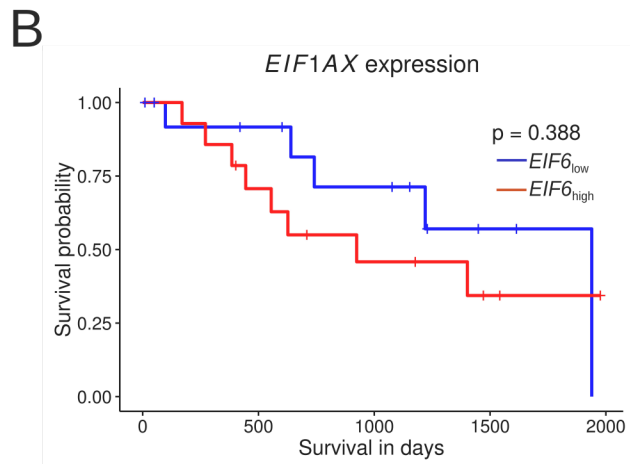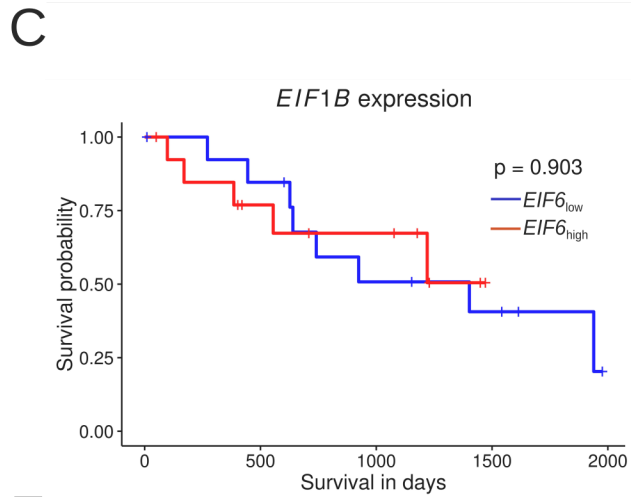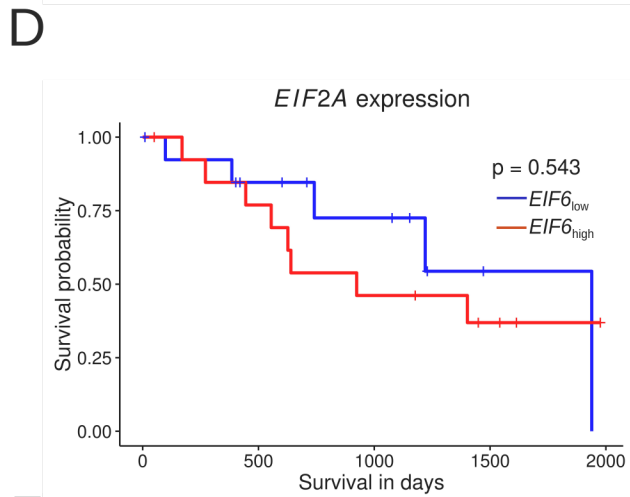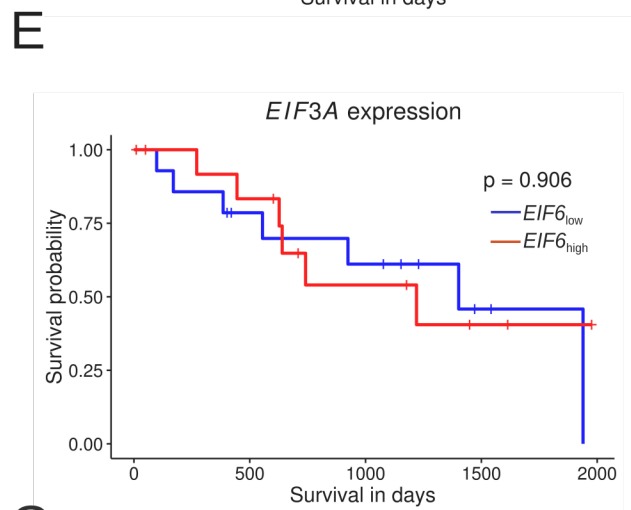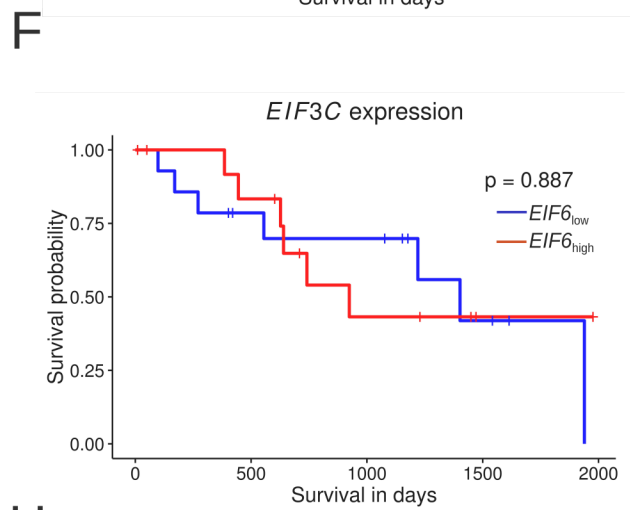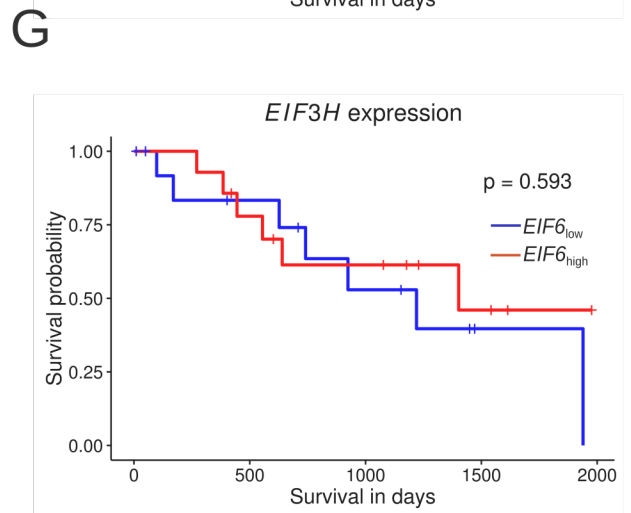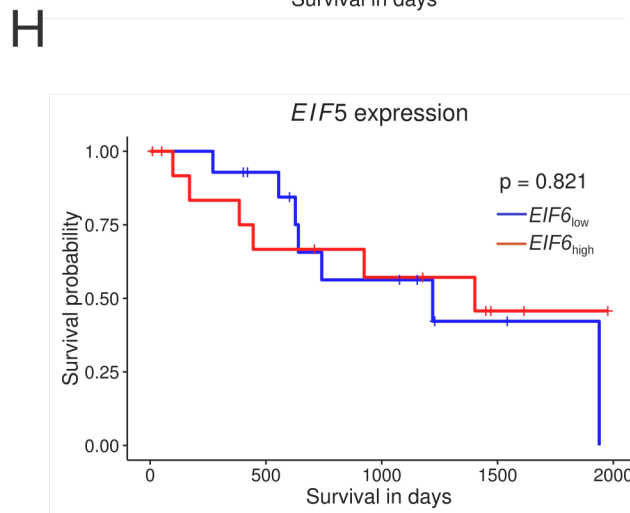

Supplement: Supplementary file 1 — Fig. S1: Clinical relevance of EIF1, EIF1AX, EIF1B, EIF2A, EIF3A, EIF3C, EIF3Hf and EIF5 gene expression on BTC patients` overall survival using TCGA data set. (A-H) Kaplan–Meier curves comparing the median EIF1 (p = 0.204), EIF1AX (p = 0.388), EIF1B (p = 0.903), EIF2A (p = 0.543), EIF3A (p = 0.906), EIF3C (p = 0.887), EIF3H (p = 0.593), and EIF5 (p = 0.821) gene expression and BTC patients´ overall survival (n = 28) of TCGA database an in silico analysis. High expression is highlighted in red and low expression in blue. (PDF 489 kb) [file 432_2019_3030_MOESM1_ESM.pdf]

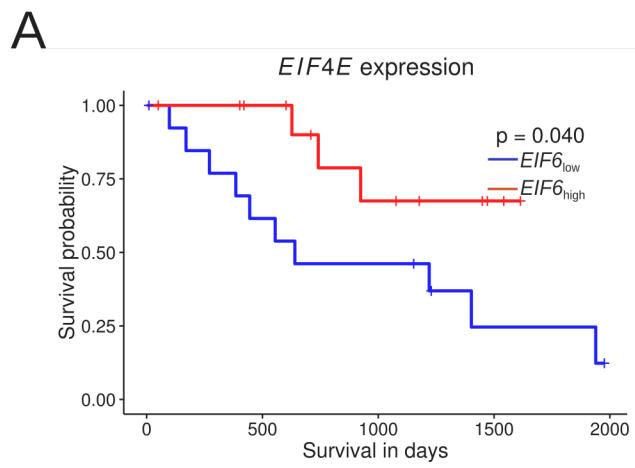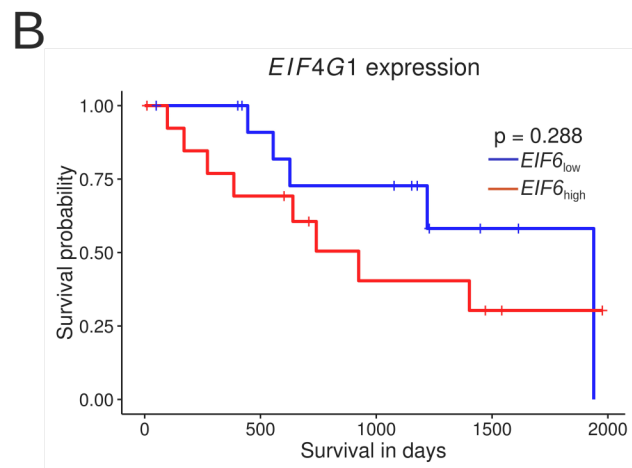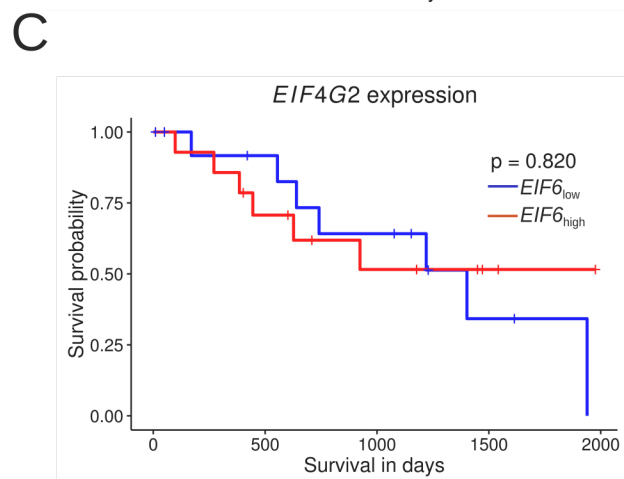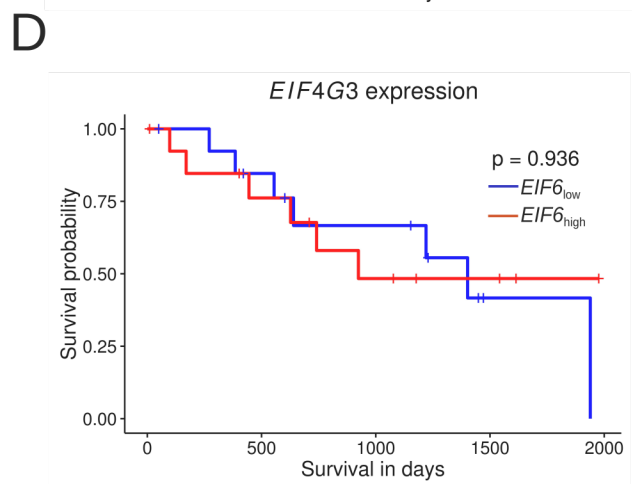

Supplement: Supplementary file 2 — Fig. S2: Clinical relevance of EIF4E, EIF4G1, EIF4G2, and EIF4G3 patients’ overall survival using TCGA data set. (A–D) Kaplan–Meier curves comparing the median EIF4E (p = 0.040), EIF4G1 (p = 0.288), EIF4G2 (p = 0.820), and EIF4G3 (p = 0.936) gene expression and overall survival of BTC patients´ of TCGA dataset an in silico analysis for BTC (n = 28). High expression is highlighted in red and low expression in blue. (PDF 248 kb) [file 432_2019_3030_MOESM2_ESM.pdf]
